# Tackle‐Based Head Injury Assessment (HIA) Risk Factors in the National Rugby League: Does the Ball Carrier's Evasion Technique or the Tackler's Head Position Influence HIA Risk?

**DOI:** 10.1002/ejsc.70138

**Published:** 2026-02-20

**Authors:** Sophie Carlton‐Greaves, Suzi Edwards, Ben Jones, Ross Tucker, Grant L. Iverson, Andrew J. Gardner

**Affiliations:** ^1^ Sydney School of Health Sciences, Discipline of Exercise & Sport Science, Faculty of Medicine & Health The University of Sydney Camperdown New South Wales Australia; ^2^ Carnegie Applied Rugby Research (CARR) Centre Carnegie School of Sport Leeds Beckett University Leeds UK; ^3^ England Performance Unit Rugby Football League Manchester UK; ^4^ Division of Physiological Sciences, Department of Human Biology, Faculty of Health Sciences The University of Cape Town Cape Town South Africa; ^5^ School of Behavioural and Health Sciences, Faculty of Health Sciences Australian Catholic University Brisbane QLD Australia; ^6^ Premiership Rugby Limited London UK; ^7^ World Rugby, Pty (Ltd) Dublin Ireland; ^8^ Department of Physical Medicine and Rehabilitation Harvard Medical School Boston Massachusetts USA; ^9^ Spaulding Rehabilitation Hospital and the Schoen Adams Research Institute at Spaulding Rehabilitation Charlestown Massachusetts USA; ^10^ Mass General for Children Sports Concussion Program Boston Massachusetts USA; ^11^ School of Medicine and Public Health College of Health, Medicine, & Wellbeing University of Newcastle Callaghan NSW Australia

**Keywords:** concussion, head impact events, injury prevention, tackle technique

## Abstract

Rugby‐style tackle techniques have been evaluated for a number of years and have traditionally focused on injury prevention and performance outcomes. Recent tackle technique research has increasingly been directed towards reduced head contact and reducing the risk for concussion. This video analysis study examined the association between the ball carrier's evasion technique and the tackler's head position and the risk for a Head Injury Assessment (HIA) in professional male rugby league players. Retrospective video analysis cohort study. Three seasons of National Rugby League (NRL) head injury assessment (HIA) events (*n* = 446) were retrospectively video coded. For comparison and the calculation of propensity, 5694 tackles that did not result in a head injury were also coded. In this study, the two variables of interest were the ball carrier's evasion technique and the tackler's head position. Propensity was calculated as HIAs per 1000 tackles. The results of this study demonstrate the highest proportion of HIAs occurred when ball carriers did not utilise an evasion strategy and when the tacklers positioned their head in front of the ball carrier, especially when combined with high‐risk evasion strategies such as jumping or shoulder charging. HIA prevention initiatives should consider tackle techniques that encourage tacklers to place their head beside the ball carrier and ball carrying strategies that utilise an evasion technique, such as twisting or bending the torso. Further tackle‐based research is required in female and youth players to evaluate whether the findings in elite male players generalise to other rugby league players.

## Highlights

- Ball carrier tackle evasion techniques appear to reduce risk of a Head Injury Assessment in professional male rugby league.
- Tackler head positioning to the side of the ball carrier reduces the risk of a Head Injury Assessment in professional male rugby league.
- A safer and more effective tackle is likely to be achieved with a ball carrier using an evasion technique and the tackler positioning their head outside of the ball carrier's body.

## 1 Introduction

By its very nature as a full‐contact collision sport, there is a risk of head contact and concussion to players during rugby league match play (Cross et al. 2019). At the elite level the incidence of potential concussions (Head Injury Assessments or HIAs) has been reported at 1.56 HIAs per 1000 tackles or a tackle HIA every 0.90 matches (A. J. Gardner et al. 2021), whereas the incidence of concussion has been reported at 14.8 concussion per 1000 player match hours (Gardner et al. 2015a; Eastwood et al. 2023). The most common rugby league match event that results in a concussion is the tackle (Gardner et al. 2015b), with the tackler being reported to have a greater risk for concussion in elite‐level men's rugby league than the ball carrier (Gardner et al. 2015b; Tucker et al. 2017; Fuller et al. 2010). In order to consider approaches for modifying tackle‐based HIA and concussion risk factors, understanding variables that result in a higher risk will enable the development of targeted interventions. Intuitively, focusing on modifying the behaviour of the tackler and the ball carrier in the pre‐contact phase of the tackle appears to be an appropriate target, as this pre‐contact phase influences HIA occurrence the most (Tierney et al. 2018).

Exploring the effect of differing ball carrier evasion strategies and tackler head position is important because these may be controllable factors in the tackle. In tackle‐based sports such as rugby league and rugby union, the evasion strategy ball carriers select and the position tacklers choose to get their head into, can impact not only the effectiveness of their carry or tackle (Speranza et al. 2017), but also the HIA and concussion risk for both the ball carrier and the tackler (Tierney et al. 2018; Sobue et al. 2017; Tierney et al. 2019; Suzuki et al. 2021). Equally, the evasion strategy of ball carriers (e.g., moving late) may influence where the tacklers put their head (Owen et al. 2025). Appropriate tackler head placement has been identified as an important aspect of any proposed tackle technique intervention, as the greatest odds of an elite‐level male rugby league tackler sustaining a concussion occurred when a tackler had his head positioned in front of the ball carrier (Owen et al. 2025). The behaviour of ball carriers in terms of the use of their leading arm (e.g., ball carrying arm fend, non‐ball carrying arm fend, bump position and hand off) was also found to be a predictor of concussion. Any type of ball carrier leading arm contact with a tackler resulted in significantly greater odds of a concussion compared to no use of the leading arm (Owen et al. 2025). Therefore, identifying the ball carrier evasion strategies and tackler head positioning that have the lowest risk of resulting in a head injury are crucial to player and team performance and injury prevention.

As such, the aim of this study was to evaluate two pre‐contact phase variables, one related to the ball carrier and one related to the tackler. This study examined the association between the risk for an HIA and the ball carrier's evasion strategy in combination with the tackler's head position, in the pre‐contact phase in professional male rugby league players.

## 2 Methods

### 2.1 Participants

An existing video review database that contains coding of game play and tackle‐based variables of professional male National Rugby League (NRL) players was used in this study (Gardner et al. 2021; Gardner et al. 2024). The database contains coded variables from three consecutive seasons (2017, 2018 and 2019). During those seasons the NRL had 16 clubs from Australia (*n* = 15) and New Zealand (*n* = 1) competing for the men's premiership. Each club plays 24 regular season games. For the clubs that finish in the top eight following the regular season a 4‐week postseason is played culminating in a Grand Final. In total there are 201 (192 regular season and 9 post‐season) games per season. In accordance with the NRL and Rugby League Players Association (RLPA) Collective Bargaining Agreement, consent for deidentified injury data to be used in this study was provided a priori by all NRL players. The study was approved by the institution’s human ethics committee.

### 2.2 Procedures

The procedures of the NRL video analysis research programme, of which this study forms a part of, have been described previously (Gardner et al. 2021; Gardner et al. 2024). To reiterate, for the first two seasons of (2017 and 2018), head impact events (resulting in an HIA) were monitored and recorded via two avenues that served as in‐game injury surveillance in the NRL during those seasons: (i) a sideline injury surveillance (SIS) system and (ii) the team medical staff. In the third season (i.e., 2019 NRL season), an additional in‐game injury surveillance process was added; the medical bunker (an offsite external facility). The NRL HIA process has also been previously described (Gardner et al. 2021). All HIAs identified by any of the three surveillance systems were recorded and uploaded to the *GamePlan* Application. The video footage on the *GamePlan* Application was reviewed by the first author, who coded all variables using a predefined coding matrix. This coding matrix was developed from work conducted in elite‐level rugby union (Cross et al. 2019; Fuller et al. 2010; McIntosh et al. 2010; Quarrie et al. 2008) and our own work in elite‐level rugby league (Gardner et al. 2017) and aligns with other rugby league frameworks (Hopkinson et al. 2022). The variables specific to this study included: the ball carrier's evasion technique and the position of the tackle's head in relation to the ball carrier in the pre‐contact phase of the tackle (see Supporting Information S1: Tables S1 and S2 for specific details on these two variables). The ball carrier evasion technique variable was operationalised as any pre‐contact behaviour that the ball carrier engages in to either evade the tackle (e.g., stepping or a hand palm) or reduce the tackler's likelihood of successfully executing the tackle (e.g., raising up forearm bumpers, bending/leaning into the contact, turning side‐on).

As described previously (Gardner et al. 2021), 5694 control tackles that did not result in concussion were coded from eight randomly selected matches without HIAs for comparison to the 472 HIAs recorded over the three NRL seasons. A tackle was defined as ‘any event where one or more tacklers attempted to stop or impede the ball carrier whether or not the ball carrier was brought to ground.’ The only exclusion criteria that applied to this study related to the quality of the video, as previously described (Gardner et al. 2021; Tucker et al. 2017). However, it was not necessary to apply any exclusion criteria to the two variables of interest in this study. In this study we use *incidence* to refer to the variables that contribute the most to the total HIA events and we use *propensity* when identifying the inherently high‐risk variables, regardless of how commonly these variables have been observed.

### 2.3 Data Analysis

The propensity of HIA events per 1000 tackles was calculated by dividing the number of HIA events occurring from the variable of interest (i.e., ball carrier evasion technique or the tackler's head position at pre‐contact) by the total number of that tackle variable of interest, obtained from the control tackles. Data are presented as means and 95% CIs, where the 95% CIs were calculated using the formula: mean +/− (SD/√n). The standard statistical package (SPSS, Version 24.0) was used for the statistical analysis in this study.

## 3 Results

### 3.1 Ball Carrier Evasion Technique

The number of events and propensity for a ball carrier HIA and a tackler HIA as a function of the ball carrier's evasion technique is shown in Table 1. When the ball carrier did not utilise an evasion method in the tackle, it accounted for 46.9% of total HIA cases, with a propensity of 2.14 (95% CI: 1.87–2.45) HIAs per 1000 tackles. The most frequent ball carrier evasion method was a leaning/bent torso into the tackle (*n* = 110, 24.7% of HIAs), although this had a relatively low propensity of 1.60 HIAs per 1000 tackles (95% CI: 1.33–1.93). Both the tackler and the ball carrier had the highest propensity for an HIA when the ball carrier used the evasion strategy of jumping 29.85 [95%: 13.41 to 66.45] and 14.93 [95% CI: 4.81 to 46.28] HIAs per 1000 tackles, respectively), although there were only nine HIA cases reported with this occurring. This is followed by ball carriers leading with their shoulder into the tackle with a propensity of 23.22 (95% CI: 11.07–48.70) HIAs per 1000 tackles for the tacklers and 3.32 (95% CI: 0.47–23.55) HIAs per 1000 tackles for the ball carriers. Evasion strategies by the ball carrier that lead to low propensity were footwork/step (0), ducked head (0.58 [95% CI: 0.22 to 1.54]) and forearm bumper (0.57 [95% CI: 0.37 to 0.88].

**TABLE 1:** Propensity for HIAs as a function of the ball carriers' evasion techniques.

Ball carrier evasion technique	All HIAs			Tackler HIAs			Ball carrier HIAs		
	HIA events	Propensity (HIAs per 1000 tackles)	95% confidence intervals	HIA events	Propensity (HIAs per 1000 tackles)	95% confidence intervals	HIA events	Propensity (HIAs per 1000 tackles)	95% confidence intervals
Jumping	9	44.78	23.30–86.06	6	29.85	13.41–66.45	3	14.93	4.81–46.28
Shoulder	8	26.53	13.27–53.06	7	23.22	11.07–48.7	1	3.32	0.47–23.55
Not applicable	7	15.48	7.38–32.47	2	4.42	1.11–17.68	5	11.06	4.60–26.56
Hand fend	34	2.16	1.54–3.03	22	1.40	0.92–2.12	12	0.76	0.43–1.34
None	209	2.14	1.87–2.45	113	1.16	0.96–1.39	96	0.98	0.80–1.20
Lean/bend torso	110	1.60	1.33–1.93	80	1.17	0.94–1.45	30	0.44	0.31–0.63
Twist/spin	20	1.26	0.81–1.95	11	0.69	0.38–1.25	9	0.57	0.29–1.09
Side‐on	23	1.24	0.82–1.87	23	1.24	0.82–1.87	0	0.00	N/A
Ducked head	4	0.58	0.22–1.54	0	0.00	N/A	4	0.58	0.22–1.54
Forearm bumper	20	0.57	0.37–0.88	17	0.48	0.30–0.78	3	0.09	0.03–0.26
Footwork/step	0	0.00	N/A	0	0.00	N/A	0	0.00	N/A
Ball bump	2	N/A	N/A	2	N/A	N/A	0	N/A	N/A
Total	446	1.56	1.87–2.45	283	0.99	0.96–1.39	163	0.57	0.80–1.20

Abbreviations: HIAs, Head Injury Assessments; N/A, not applicable.

### 3.2 Tackler's Head Position

Table 2 presents the number of HIA events and propensity for a ball carrier HIA and a tackler HIA as a function of the tackler's head position. With regard to the head position of the tackler, the highest HIA propensity was observed when the tackler's head was positioned in front of the ball carrier (*n* = 276, 5.30 HIAs per 1000 tackles; 95% CI: 4.71–5.96), accounting for 61.95% of HIAs. When the tackler's head was positioned in front of the ball carrier, both the tackler (*n* = 162; propensity for an HIA 3.11 HIAs per 1000 tackles; 95% CI: 2.67–3.63) and the ball carrier (*n* = 114; propensity for an HIA 2.19 HIAs per 1000 tackles; 95% CI: 1.82–2.63) had the highest frequency. While the second most frequent overall HIAs occurred when tacklers placed their head beside the ball carriers (*n* = 84), this head position led to a low propensity overall (0.53 HIAs per 1000 tackles; 95% CI: 0.43–0.66) for the tackler (*n* = 51; 0.32 HIAs per 1000 tackles; 95% CI: 0.24–0.42) and for the ball carrier (*n* = 33; 0.21 HIAs per 1000 tackles; 95% CI: 0.15–0.30).

**TABLE 2:** Propensity for HIAs as a function of the tackler's head position in relation to the ball carrier.

Tackler head position	All HIAs			Tackler HIAs			Ball carrier HIAs		
	HIA events	Propensity (HIAs per 1000 tackles)	95% confidence intervals	HIA events	Propensity (HIAs per 1000 tackles)	95% confidence intervals	HIA events	Propensity (HIAs per 1000 tackles)	95% confidence intervals
In front	276	5.30	4.71–5.96	162	3.11	2.67–3.63	114	2.19	1.82–2.63
Behind	68	1.82	1.43–2.31	62	1.66	1.29–2.13	6	0.16	0.07–0.36
Beside	84	0.53	0.43–0.66	51	0.32	0.24–0.42	33	0.21	0.15–0.30
Above	10	0.26	0.14–0.48	6	0.16	0.07–0.36	4	0.10	0.04–0.27
Not coded	8	N/A	N/A	2	N/A	N/A	6	N/A	N/A
Total	446	1.56	1.43–2.30	283	0.99	0.88–1.11	163	0.57	0.49–0.66

Abbreviations: HIAs, Head Injury Assessments; N/A, not applicable.

### 3.3 Interaction of the Ball Carrier Evasion Technique and the Tackler's Head Position

The interaction between the ball carrier's evasion technique and the tackler's head position in the frequency and propensity for HIAs for the ball carrier and a tackler HIA is shown in Table 3. HIAs most commonly occurred for both the tackler and the ball carrier when the ball carriers did not utilise an evasion method and the tacklers positioned their head directly in front of the ball carrier (*n* = 136, 31% of HIAs), with a higher propensity of 3.97 HIAs per 1000 tackles (95% CI: 3.36–4.70) compared to when the tacklers positioned their head bedside the ball carriers (0.89 HIAs per 1000 tackles, 95% CI: 0.64–1.23). The highest propensity for HIAs for either the tackler or ball carrier was when tacklers placed their head in front of the ball carriers combined with the ball carriers employing an evasion strategy of jumping (99.50 HIAs per 1000 tackles; 95% CI: 41.42 to 239.06; *n* = 5) or leading with a shoulder in to the tackle (79.60 HIAs per 1000 tackles; 95% CI: 39.81 to 159.17; *n* = 8).

**TABLE 3:** Number of HIAs and propensity as a function of the ball carrier's evasion techniques in interaction with the tackler's head position.

	None	Hand fend	Forearm buffer	Shoulder	Ball bump	Lean/bend torso	Twist/spin	Side‐on	Footwork/step	Ducked head	Jumping
	Propensity (n)	Propensity (n)	Propensity (n)	Propensity (n)	Propensity (n)	Propensity (n)	Propensity (n)	Propensity (n)	Propensity (n)	Propensity (n)	Propensity (n)
All HIAs											
Above	5.82 (12)	3.98 (1)	0.83 (1)	0.00 (0)	0.00 (0)	2.21 (1)	0.00 (0)	0.00 (0)	0.00 (0)	0.00 (0)	0.00 (3)
In front	3.97 (136)	2.80 (27)	0.85 (16)	79.60 (8)	0.00 (1)	2.61 (68)	1.64 (13)	1.48 (14)	0.00 (0)	1.99 (5)	99.50 (5)
Behind	2.08 (16)	0.83 (1)	0.00 (0)	0.00 (0)	0.00 (0)	7.46 (3)	2.84 (1)	0.00 (0)	0.00 (0)	0.00 (0)	0.00 (1)
Beside	0.89 (26)	1.40 (5)	0.24 (3)	0.00 (0)	0.00 (1)	0.99 (37)	1.01 (6)	1.20 (9)	0.00 (0)	0.00 (0)	0.00 (0)
Tackler HIAs											
Above	3.88 (8)	3.98 (1)	0.83 (1)	0.00 (0)	0.00 (0)	0.00 (0)	0.00 (0)	0.00 (0)	0.00 (0)	0.00 (0)	0.00 (2)
In front	1.69 (58)	0.69 (15)	0.69 (13)	69.65 (7)	0.00 (1)	1.61 (42)	0.88 (7)	1.48 (14)	0.00 (0)	0.00 (0)	59.70 (3)
Behind	1.69 (13)	0.83 (1)	0.00 (0)	0.00 (0)	0.00 (0)	7.46 (3)	0.00 (0)	0.00 (0)	0.00 (0)	0.00 (0)	0.00 (0)
Beside	0.74 (30)	1.40 (5)	0.24 (3)	0.00 (0)	0.00 (1)	0.91 (34)	0.67 (4)	1.20 (9)	0.00 (0)	0.00 (0)	0.00 (0)
Ball carrier HIAs											
Above	1.94 (4)	0.00 (0)	0.00 (0)	0.00 (0)	0.00 (0)	2.21 (1)	0.00 (0)	0.00 (0)	0.00 (0)	0.00 (0)	0.00 (1)
In front	2.28 (78)	1.24 (12)	0.16 (3)	9.95 (1)	0.00 (0)	1.00 (26)	0.76 (6)	0.00 (0)	0.00 (0)	1.99 (4)	39.80 (2)
Behind	0.39 (3)	0.00 (0)	0.00 (0)	0.00 (0)	0.00 (0)	0.00 (0)	2.84 (1)	0.00 (0)	0.00 (0)	0.00 (0)	0.00 (0)
Beside	0.15 (6)	0.00 (0)	0.00 (0)	0.00 (0)	0.00 (0)	0.08 (3)	0.34 (2)	0.00 (0)	0.00 (0)	0.00 (0)	0.00 (0)

Abbreviation: HIAs, Head Injury Assessments.

The third highest propensity for a tackler sustaining an HIA occurred when tacklers positioned their head behind the ball carrier and when the ball carriers lean/bend their torso as an evasion strategy (7.46 HIAs per 1000 tackles; 95%: 2.41–23.24). If tacklers position their head beside the ball carriers when the ball carriers lean/bend their torso into a tackle they decrease their propensity of HIA to 0.99 HIA per 1000 tackles (95% 0.72–1.37) and the ball carrier propensity of HIA to 0.08 HIA per 1000 tackles (95% 0.03–0.25). The hand fend was the most common evasion strategy employed by the ball carrier (3.98 HIAs per 1000 tackles; 95%: 0.56–28.26). When ball carriers utilised a twist/spin evasion strategy their propensity for HIA increased when the tacklers placed their head beside (0.34 HIA per 1000 tackles; 95% CI: 0.08–1.5) to behind the ball carrier (2.84 HIAs per 1000 tackles; 95%: 0.40–20.18).

## 4 Discussion

The current study further expanded on our programme of research characterising tackle‐based risk factors in the National Rugby League (Gardner et al. 2021). Here we have identified that the ball carrier's evasion technique and the tackler's head position prior to contact influence the incidence and propensity of an HIA. The highest incidence of HIA occurred when the ball carrier did not employ any evasion technique and the highest propensity for HIAs occurred when the ball carrier used the evasion strategies of jumping or leading with the shoulder. Tacklers who positioned their head in front of the ball carrier had the highest incidence and propensity for HIAs.

### 4.1 Ball Carrier Evasion Technique

In the NRL, the ball carrier can adopt a variety of evasion techniques to optimise performance (Connor et al. 2018) and alter the risk for an HIA (Lang et al. 2024). Selection of the evasion strategy is vital as 59% of the total ball carrier HIAs occurred when the ball carrier did not utilise a tackle evasion technique. Both the ball carrier and the tackler are at a lower risk of an HIA if the ball carrier uses an evasion technique. This finding is consistent with previous research findings (Lang et al. 2024). The purpose of a ball carrier using the forearm bumper evasion strategy is to create more space between the tackler and ball carrier, which in turn reduces the head‐on‐head contact risk. This occurs because the player's heads are further apart from each other in contact. The data from the current study reveals the lowest propensity for HIA for both the tackler and the ball carrier occurs when the ball carrier uses the forearm bumper evasion strategy. Aside from using no evasion technique at all, the ball carrier's next most employed evasion technique was leaning/bending the torso or utilising a twist/spin movement. These techniques were successful from an HIA risk perspective (they have required a relatively low HIA propensity) and require little footwork skills or agility, as they mainly require an upper body movement. It was noted that the HIA propensity for these ball carrier evasion strategies was higher for the tackler than the ball carrier. This may be because the tackler movement is a reactionary movement, as opposed to planned movement, but this will require further investigation in future research. The tackler may take more time to process the visual information of the ball carrier movements and then attempt to effectively react to the ball carrier's body position. Jumping in rugby league is a rarely performed movement with only 13 jumping events as an evasion strategy recorded in this study. Most players are taught from a very young age not to jump into a tackle. The current study supports this coaching advice. When a ball carrier jumped as the evasion strategy, it resulted in the highest HIA propensity, with 9 out of these 13 jumping cases resulting in an HIA. Jumping in general is difficult to mitigate against as jumping plays a key part in aerial contests and winning possession off a kick, but it is important players are aware of the HIA risk when performing these jumps. Similarly, slipping/falling has a high propensity, but this is also difficult to mitigate against, due to their usual unpredictable and accidental nature. While the shoulder charge tackle technique has now been outlawed, (NRL Judiciary and Match Review n.a.) ball carriers can still legally lead into contact with their shoulder. When this occurs, the ball carrier's shoulder applies an external force to the tackler over a very short duration (i.e., higher impulse) creating an effective performance strategy to evade the tackler. The tacklers must react quickly to the ball carriers in order to avoid their shoulder making effective contact that can result in a missed tackle (a positive performance outcome for the ball carrier but a negative performance outcome for the tackler) or direct contact with the head and a risk for an HIA or concussion. Although this can be an effective evasion strategy for the ball carrier, it poses a high HIA propensity (26.53) due to the shoulder being in close proximity to the tackler's head, thus ball carriers leading into contact with their shoulder may be a target for rule modification, to reduce the HIA and concussion risk.

### 4.2 Tacklers Head Position

The tackler's head position when engaging in contact is recommended to be positioned beside the ball carrier's body as opposed to in front (Lang et al. 2024). The results of this study have revealed that when the tackler's head is positioned beside the ball carrier's body the HIA propensity was reduced by 90%, compared to the tackler's head position being positioned in front of the ball carrier's body. Tacklers in this study who position their head in front of the ball carrier have the highest frequency and propensity of HIAs, with 61% of HIAs occurring when the tackler's head was positioned in front of the ball carrier's body. Although having your head behind and above the ball have the lowest propensity, these head positions are not usually an option when attempting to make most tackles during an NRL game, especially not for front‐on tackles. Only when a ball carrier has a very low stance when entering the tackle, would a tackler be able to get over the top of them. Historically, rugby league players have been coached to execute lower body tackles using a ‘cheek‐on‐cheek’ technique, where they have been instructed to aim to get their head on the ball carrier's upper thigh/glute area. This technique positions the tackler's head in close proximity with the ball carrier's hip, which is a high‐risk area. A safer area to aim for would be the torso, as it is softer (Fuller et al. 2010). Tackle technique education may be an effective intervention strategy, even at the elite level.

### 4.3 Interaction of the Ball Carrier Evasion Technique and the Tackler's Head Position

The tackle is a complex game play event that requires tacklers to use narrow external and internal attentional focus (Magill et al. 2018) to effectively adjust their body and head position based on cues from the ball carrier's evasion strategy, to execute a safer and effective tackle. This has been highlighted by previous research, which suggests that ‘identify/tracking the ball carrier’ is an effective way of reducing HIA propensity as it allows for tacklers to individualise their tackling technique based on the stimulus in front of them (Tierney et al. 2018). Evaluating the interaction between the player's head position and the ball carrier's evasion strategy highlighted that the highest incidence of HIA occurred when the ball carriers did not utilise an evasion method and the tacklers positioned their heads in front of the ball carrier. The frequency of HIAs was highest for all evasion strategies when the tackler's head was in front of the ball carrier, suggesting that if tacklers can use narrow internal attentional focus to devise a plan to position their head beside the ball carrier, then that will decrease the HIA propensity for the tackler, regardless of what evasion strategy the ball carrier selects. For example, no tacklers were at risk of an HIA from ball carriers leading with their shoulder into the tackle if the tackler's head was not positioned in front of the ball carrier. In general, the tackler is at a higher risk of HIA than the ball carrier (Gardner et al. 2015b), further supporting the importance for tacklers to use their attentional focus to adjust their tackle plan and execution based on the ball carrier's movement and evasion strategy.

## 5 Limitations

There are a number of limitations to this study and a broader research programme that have been previously highlighted (Gardner et al. 2021). The subjective nature of the qualitative approach to interpretation and coding of tackle variables may create rater bias as a single analyst coded all variables within this study. This single rater approach has the advantage of avoiding any considerations of between‐rater differences, but the subjectivity of coding these variables represents a potential source of error in the analysis. Specific tackle characteristics have been analysed discretely, however, all tackle variables interact with one another, as such our approach does not consider the dynamic and complex nature of the tackle and how multiple factors may affect risk in subtle ways. Third, the generalisability of these results to other levels of competitions, age or women players is unknown.

## 6 Conclusion

It is a high priority for contact and collision sports to reduce the risk of head contact and potential concussion for athletes, particularly during match play. In a tackle‐based sport such as rugby league, focusing on modifying the behaviour of the tackler and the ball carrier in the pre‐contact phase of the tackle appears to be a promising intervention target for reducing these risks. In this video analysis study both the ball carrier's evasion strategy and the tackler's head position were found to significantly influence the risk of an HIA in male professional rugby league match play. We identified that the absence of an evasion strategy and tacklers positioning their head in front of the ball carrier were the most frequent contributors to HIA incidents. High‐risk evasion techniques such as jumping or leading with the shoulder charges, though less common, were associated with the highest HIA propensities—particularly for tacklers.

These findings reinforce the need for injury prevention initiatives to focus on teaching effective ball‐carrying evasion techniques and encouraging tacklers to adopt safer head positions during contact. Integrating attentional focus training and modifying traditional tackling cues may further reduce HIA risk and improve rugby league player safety.

## 7 Practical Implications

- These results provide further evidence to support a coaching focus on ball carrier evasion and tackle techniques to reduce the risk for an HIA.
- Educating players to adapt their tackling technique based off the ball carriers' approach and evasion strategy will allow for a safer and more effective tackle.
- Interventions through game or rule modification that may directly address ball carrier evasion strategies and tackle techniques may also be effective.

## Conflicts of Interest

The authors declare no conflicts of interest.

## Disclosure

Suzi Edwards leads a tackle re‐education program that is partially funded by a NHMRC Ideas 2021 Grant (202718). This NHMRC Ideas 2021 grant financially supports the PhD Scholarship of Oscar Stelzer‐Hiller. Ken Quarrie is employed as the senior sport scientist for New Zealand Rugby. Grant Iverson serves or has served as a scientific advisor for NanoDX, Sway Operations, LLC and Highmark Inc., He has a clinical and consulting practice in forensic neuropsychology, including expert testimony, involving individuals who have sustained mild TBIs (including former athletes). He has received past research support or funding from several test publishing companies, including ImPACT Applications Inc., CNS Vital Signs, and Psychological Assessment Resources (PAR Inc.). He has received research funding as a principal investigator from the National Football League and subcontract grant funding as a collaborator from the Harvard Integrated Program to Protect and Improve the Health of National Football League Players Association Members. He acknowledges unrestricted philanthropic support from the Mooney‐Reed Charitable Foundation, ImPACT Applications Inc. National Rugby League, the Heinz Family Foundation, Boston Bolts and the Schoen Adams Research Institute at Spaulding Rehabilitation. None of the above entities were involved in the study design, analysis, interpretation, the writing of this article, or the decision to submit it for publication. Andrew Gardner Ph.D. has a clinical practice in neuropsychology involving individuals who have sustained sport‐related concussion. He is a concussion consultant to Rugby Australia and the Sydney Swans Football Club. He is the global clinical lead for the World Rugby Brain Health Service. He is a member of the World Rugby Concussion Working Group, and a member of the Australian Football League Concussion Scientific Advisory Committee. He has received travel funding or been reimbursed by professional sporting bodies and commercial organisations for discussing or presenting sport‐related concussion research at meetings, scientific conferences, workshops and symposiums. He is supported by a National Health and Medical Research Council (NHMRC) Investigator Grant. He acknowledges unrestricted philanthropic support from the National Rugby League, the International Olympic Committee, the Australian Sports Commission and World Rugby for research in former elite level athletes.

## Supporting information


Supporting Information S1

## Data Availability

The data that support the findings of this study are available from the corresponding author upon reasonable request.
