# Supplementary material for: Tackle‐Based Head Injury Assessment (HIA) Risk Factors in the National Rugby League: Does the Ball Carrier's Evasion Technique or the Tackler's Head Position Influence HIA Risk?
Source: Eur J Sport Sci. 2026 Feb 20;26(3):e70138. doi: 10.1002/ejsc.70138 (PMC12927997; doi:10.1002/ejsc.70138)
Supplement: Supplementary file 1 — Supporting Information S1 [file EJSC-26-e70138-s001.docx]

**Supplementary Material**

Supplementary Table 1. Variable labels and definitions.

| Variable label | Coding Description |
| --- | --- |
| Ball Carrier Evasion Technique | The strategy used by the ball carrier to avoid or to brace for contact. |
| Tackler Head Position | The head position of the tackler relative to the ball carrier’s body. |

Adapted from Gardner AJ, Iverson GL, Bloomfield P, et al. SCRIMMAGE for Brain Health in the NRL (Studying Contact Replays: Investigating Mechanisms, Management, and Game Exposures in the Australasian National Rugby League): A protocol for a database design. *BMJ Open Sport Exerc Med* 2024; 10(4):e002216.

Supplementary Table 2. Variable codes.

| Variable label | Coding Description |
| --- | --- |
| Ball Carrier Evasion Technique | None: the ball carrier did not employ any evasion technique.  Hand fend: the ball carrier put out their non-ball caring hand to push the tackler away.  Forearm bumpers: the ball carrier held the ball in two hands into contact and used their forearms as the first point of contact.  Shoulder: the ball carrier turned and used their shoulder as the first point of contact.  Ball Bump: the ball carrier used the ball as the first point of contact.  Lean or bend torso: the ball carrier leans forward and bends into contact.  Twist or spin: the ball carrier hits and twists or spins at the point of contact.  Side-on: the ball carrier turns side on into contact.  Footwork or step: the ball carrier changes direction in to contact by using footwork.  Ducked head: the ball carrier bends down lowering and leading with their head into contact.  Jumping: the ball carrier jumps up into contact. |
| Tackler Head Position | In front: the tackler’s head was in front of the ball carrier’s body.  Side: meaning outside the ball carrier’s body.  Behind: the tackler’s head was behind the ball carrier’s body (for example, a tackler coming in from the side, often put their head behind the ball carrier).  Above: the tackler’s head was above the ball carrier’s body (most often occurred when a ball carrier ducked their head into contact or slipped prior to contact). |

Adapted from Gardner AJ, Iverson GL, Bloomfield P, et al. SCRIMMAGE for Brain Health in the NRL (Studying Contact Replays: Investigating Mechanisms, Management, and Game Exposures in the Australasian National Rugby League): A protocol for a database design. *BMJ Open Sport Exerc Med* 2024; 10(4):e002216.
